# Spontaneous esophageal rupture following perforated peptic ulcer: a report of two cases

**DOI:** 10.1186/s13019-021-01431-z

**Published:** 2021-03-26

**Authors:** Chieh-Wei Chang, Yu-Ju Hung, Chien-Pin Chan, Chang-Lun Huang

**Affiliations:** 1 Division of Thoracic Surgery, Department of Surgery, Changhua Christian Hospital, No. 135 Nanxiao St., Changhua City, Changhua County 500 Taiwan; 2 Division of General Surgery, Department of Surgery, Changhua Christian Hospital, No. 135 Nanxiao St., Changhua City, Changhua County 500 Taiwan

**Keywords:** Boerhaave’s syndrome, Spontaneous esophageal rupture, Perforated peptic ulcer

## Abstract

**Background:**

Spontaneous esophageal rupture, also called Boerhaave’s syndrome, is relatively uncommon but may result in high morbidity and mortality. Synchronous presentation of spontaneous esophageal rupture and perforated peptic ulcer was rare and may contribute to the difficulty of achieving a correct diagnosis.

**Case presentation:**

We reported two patients with spontaneous esophageal rupture following perforated peptic ulcer. Both patients were successfully treated with thoracoscopic primary repair of esophageal rupture. The first patient underwent peptic ulcer repair via laparotomy. The second patient underwent laparoscopic duodenorrhaphy. Both patients resumed oral intake smoothly and were discharged uneventfully.

**Conclusion:**

Minimally invasive approaches are safe and feasible for both esophageal rupture and perforated peptic ulcer in patients diagnosed within 24 h and without shock.

## Background

Spontaneous esophageal rupture, also called Boerhaave’s syndrome, is relatively uncommon but may result in high morbidity and mortality. The classic presentations are chest or abdominal pain, shortness of breath, and shock after forced emesis or cough. Missed diagnosis as myocardial infarction, pancreatitis, and peptic ulcer perforation were reported due to similarities in symptoms [1]. Pneumomediastinum or hydropneumothorax with discontinuous esophageal wall seen on computed tomography study may hint for correct diagnosis. The primary layer-by-layer repair of injured esophagus following meticulous removal of the devitalized tissue, minimizing contamination by decortication of thoracic empyema, sepsis control, and nutritional support are all keys to success. Synchronous presentation of spontaneous esophageal rupture and perforated peptic ulcer may contribute to the difficulty of achieving a correct diagnosis, and only a few cases have been reported in the literature [2–4]. We reported two patients with spontaneous esophageal rupture following perforated peptic ulcer. Both patients were successfully treated with thoracoscopic primary repair of the ruptured esophagus and closure of peptic ulcer by laparoscopy or laparotomy.

## Case 1

**Fig. 1:**
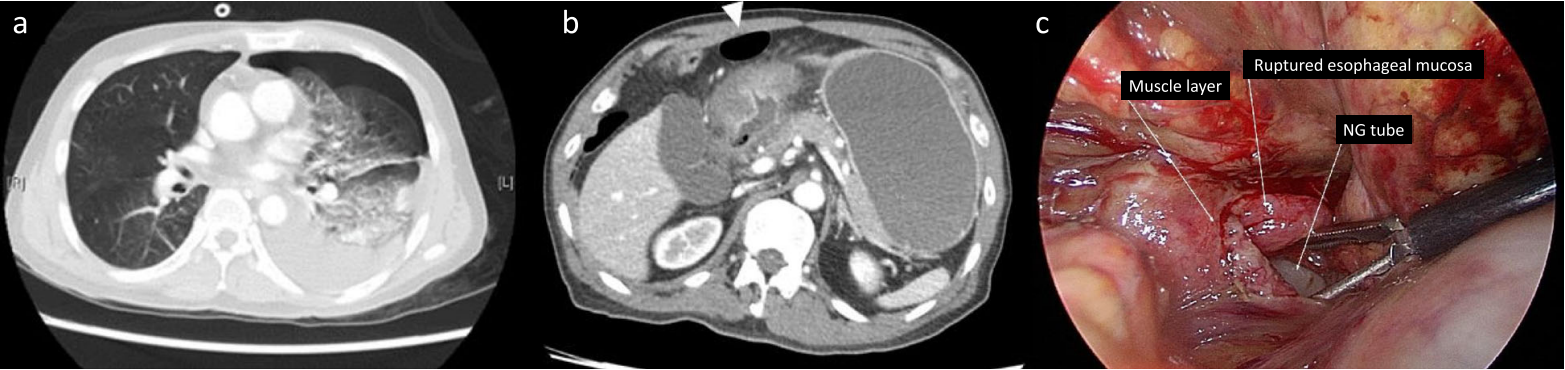
**a** Left side hydropneumothorax. **b** Pneumoperitoneum around pyloric region (white arrow head). **c** Thoracoscopic findings before repair showed ruptured esophagus with NG tube exposure

A 67-year-old male with history of lung adenocarcinoma and brain metastasis suffered from intermittent epigastric pain for several days before presentation. A sudden onset of sharp left chest pain was noted after several episodes of cough. He was brought to the Emergency Department of Changhua Christian Hospital, Taiwan. Initial physical examinations revealed sinus tachycardia without abnormal rhythm and abdominal tenderness with rebound pain. Acute myocardial infarction was initially excluded based on the electrocardiogram findings (absence of diagnostic wave change) and laboratory data (troponin I level, < 0.017 ng/mL; creatine kinase-MB mass, 0.87 ng/mL; both were within the normal range). Chest and abdominal computed tomography findings revealed left-sided hydeopneumothroax and mural wall swelling of the lower third of the esophagus with suspicion of perforation. Furthermore, distended stomach and pneumoperitoneum were also noted with a soft tissue around the pyloric region (Fig. 1a-b). A tentative diagnosis of an esophageal rupture with suspicion of peptic ulcer perforation was made. Emergent left side thoracoscopy was performed which showed turbid pleural fluid with food content. A 3-cm long rupture over the lower third of the esophagus was also observed. After evacuating the turbid pleural effusion and irrigating the pleural space, the muscle layer and mucosal layer of the esophagus were identified and repaired layer by layer thoracoscopically (Fig. 1c). No air leakage was found during the under-water test. There were two chest drains placed. Thereafter, exploratory laparotomy was performed, and a 2-cm perforated gastric ulcer over the antrum was discovered. Gastrorrhaphy and feeding jejunostomy were performed. The patient was admitted to the intensive care unit for further sepsis control and nutritional support. Enteral nutrition was resumed via jejunostomy on postoperative day 2. Esophagography was achieved on postoperative day 10 without evidence of leakage. Oral intake was then resumed, and the patient was uneventfully discharged on postoperative day 14.

## Case 2

**Fig. 2:**
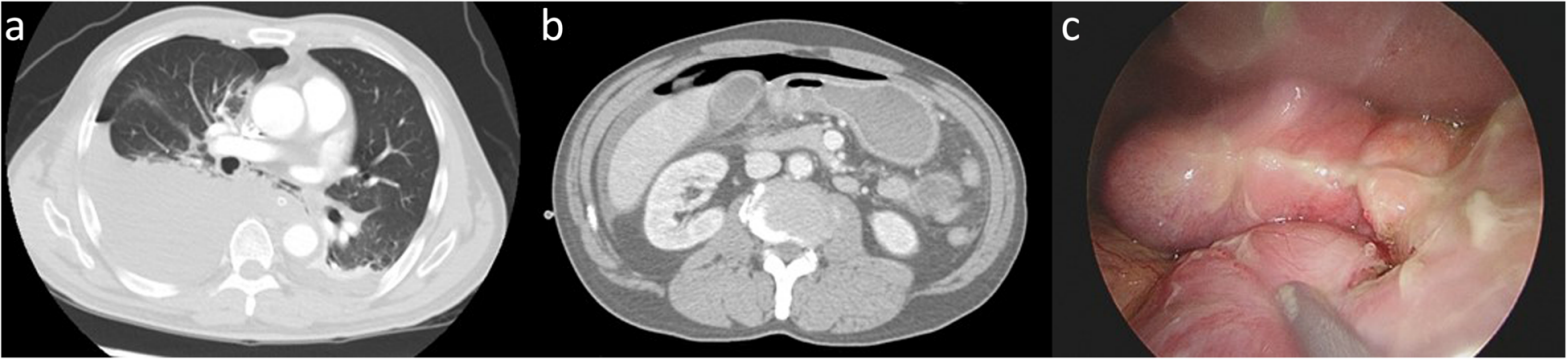
**a** Right side hydropneumothorax with mediastinal fluid accumulation. **b** Pneumoperitoneum. **c** Laparoscopy showed perforated duodenal ulcer

A 62-year-old male with history of left middle cerebral artery infarction and right hemiparesis presented with hematemesis following dyspnea, fever, and chills. He was rushed to the emergency department, and his laboratory data revealed leukocytosis with left shift (white blood cell count, 10,690/uL; neutrophil count, 68.0%) and an elevated procalcitonin level of 14.13 ng/mL. Chest computed tomography revealed a right-sided hydropneumothorax with mediastinal fluid accumulation around the lower thoracic esophagus. Additionally, pneumoperitoneum was also noted (Fig. 2a-b). An upper gastrointestinal endoscopy performed in the operation theater showed a 2-cm long transmural rupture of the lower third esophagus. Right side thoracoscopy was then performed, and the pleural space was decontaminated. A layer-by-layer thoracoscopic primary repair of the esophageal rupture was done. After turning the patient to a supine position, laparoscopy was performed which showed a 2 × 1.5-cm perforation at the first portion of the duodenum (Fig. 2c). Laparoscopic duodenorrhaphy, decompressive gastrostomy, and feeding jejunostomy were then performed after the removal of contaminated ascites. After operation, nutrition support was kept via feeding jejunostomy, and infection was controlled with antibiotics. Esophagography performed on postoperative day 11 showed no evidence of leakage. After initiating oral intake without any specific discomfort, the patient was discharged on postoperative day 16.

## Discussion and conclusions

The management for spontaneous esophageal rupture includes conservative treatment, endoscopic treatment, and surgical treatment. The factors that determine the treatment choice include the size of perforation; time interval between perforation and presentation; general condition, including shock and nutrition status; and physicians’ or surgeons’ experience and perspectives. De Schipper et al. [1] reviewed the literature before 2008 and suggested endoscopic treatment for patients diagnosed within 48 h without sepsis. Thoracotomy and primary repair should be performed in patients with sepsis, and when a patient is diagnosed after 48 h, conservative treatment should be first step. Cho et al. [5] compared primary repair in patients with Boerhaave’s syndrome using the thoracoscopy and thoracotomy approaches. The thoracoscopic group had a significantly lower operation time and fewer ventilator hours than the thoracotomy group; however, there was no difference in the length of hospital stay. Postoperative complications included pneumonia, leakage, or dysphagia. Our two patients were diagnosed within 24 h without septic shock. The esophageal ruptures were successfully treated with thoracoscopic debridement and primary repair without leakage.

Both Boerhaave’s syndrome and perforated peptic ulcer are life-threatening diseases. Only three cases have been reported in the literature, and most of them were managed by resection and reconstruction via laparotomy [2–4]. Our first patient underwent a successful thoracoscopic repair of esophageal rupture. Exploratory laparotomy was performed under general surgeon’s perspectives. After meticulous identification and evaluation on the perforation, simple closure with omental patch was performed. Our second patient was the first reported case of minimally invasive approaches for both esophageal rupture and perforated peptic ulcer in the literature. With adherence to the concepts of adequate debridement and drainage, layer-by-layer repair of esophageal rupture, and enteral nutrition route establishment, minimally invasive approaches were safe and feasible for both esophageal rupture and perforated peptic ulcer.

This report concluded that spontaneous esophageal rupture following perforated peptic ulcer is uncommon but can be life threatening. This differential diagnosis should be kept in mind, and associated examinations should be performed. Minimally invasive approaches are safe and feasible for both esophageal rupture and perforated peptic ulcer in patients diagnosed within 24 h and without shock.

## Data Availability

Not applicable.

## References

[1] de Schipper JP, Pull ter Gunne AF, Oostvogel HJ, van Laarhoven CJ (2009). Spontaneous rupture of the oesophagus: Boerhaave's syndrome in 2008. Literature review and treatment algorithm. Dig Surg.

[2] Altorjay A, Szilagyi A, Sarkany A (2005). Synchronous spontaneous perforation of the esophagus and a duodenal ulcer. Dis Esophagus.

[3] Sidhu S, Curran S, Robinson G (1998). Spontaneous oesophageal perforation with simultaneous perforated duodenal ulcer. Aust N Z J Surg.

[4] O'Neal JF (1994). A spontaneous esophageal perforation and duodenal ulcer perforation resulting in a subpulmonic abscess. W V Med J.

[5] Cho JS, Kim YD, Kim JW, I HS, Kim MS (2011). Thoracoscopic primary esophageal repair in patients with Boerhaave's syndrome. Ann Thorac Surg.

